# Influence of donor–recipient sex on engraftment of normal and leukemia stem cells in xenotransplantation

**DOI:** 10.1002/hem3.80

**Published:** 2024-05-21

**Authors:** Syed A. Mian, Linda Ariza‐McNaughton, Fernando Anjos‐Afonso, Remisha Guring, Sophie Jackson, Aytug Kizilors, John Gribben, Dominique Bonnet

**Affiliations:** ^1^ Haematopoietic Stem Cell Lab The Francis Crick Institute London UK; ^2^ Laboratory for Molecular Haemato‐Oncology King's College Hospital London London UK; ^3^ Department of Haemato‐Oncology, Barts Cancer Institute Queen Mary University of London London UK

## Abstract

Immunodeficient mouse models are widely used for the assessment of human normal and leukemic stem cells. Despite the advancements over the years, reproducibility, as well as the differences in the engraftment of human cells in recipient mice remains to be fully resolved. Here, we used various immunodeficient mouse models to characterize the effect of donor–recipient sex on the engraftment of the human leukemic and healthy cells. Donor human cells and recipient immunodeficient mice demonstrate sex‐specific engraftment levels with significant differences observed in the lineage output of normal CD34^+^ hematopoietic stem and progenitor cells upon xenotransplantation. Intriguingly, human female donor cells display heightened sensitivity to the recipient mice's gender, influencing their proliferation and resulting in significantly increased engraftment in female recipient mice. Our study underscores the intricate interplay taking place between donor and recipient characteristics, shedding light on important considerations for future studies, particularly in the context of pre‐clinical research.

## Graphical abstract

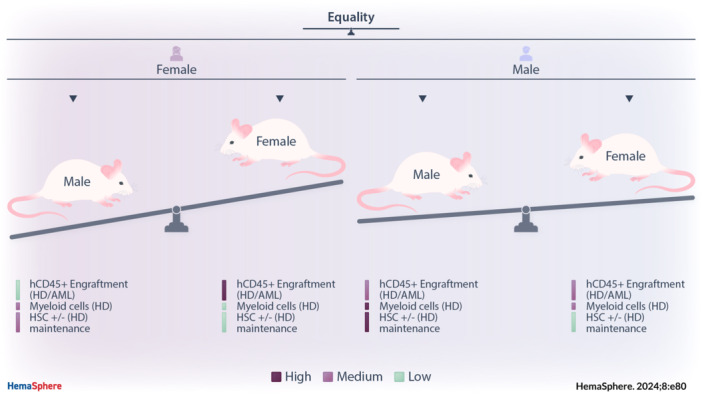

## INTRODUCTION

Human hematopoiesis involves a highly regulated set of developmental stages which at a steady state is initiated from hematopoietic stem cells (HSCs) that reside in the bone marrow (BM) tissue. These HSCs via a continuum process produce hematopoietic progenitor cells leading to the development of lineage‐restricted mature cells that perform the diverse functions of the hematopoietic system. This process is very dynamic and is controlled not only by cell‐intrinsic factors but also extracellular cues including cytokines, hormones, and infections, to maintain homeostasis in the BM and peripheral blood (PB). Together, these homeostatic mechanisms maintain the delicate balance between quiescence, self‐renewal, and differentiation in HSCs. Dysregulation at the genetic (and/or epigenetic) level occurring at the early stages of hematopoiesis can disrupt these processes and cause hematological malignancies, such as acute myeloid leukemia (AML).^1^, ^2^, ^3^, ^4^

A number of differences in hematopoiesis have been associated with biological sex, which are thought, at least in part, to be due to different hormonal profiles. Changes in hormone levels during puberty and the menstrual cycle can influence levels of certain blood cells. Androgens, such as danazol, have been used clinically to treat BM failure syndrome. Differences between men and women in the incidence, manifestation, and outcome of many hematopoietic malignancies are well recognized.^5^, ^6^, ^7^

Xenotransplantation assays using immunodeficient mouse models are routinely performed in biomedical research to identify human long‐term HSCs, assess the frequency of human leukemia‐initiating cells, test targeted therapies, vaccines, and immunotherapies, as well as investigate the function of specific genes or cell types. Several different animal models have been developed over the years but the more promising to date are the NSG (nonobese diabetic/severe combined immunodeficiency, gamma chain null mice), NSG‐SGM3 (NSG humanized with SCF, GM‐CSF, and IL‐3), and more recently the NSBGW (NSG with ckit^W41^ mutation) mice. NSG mice have been observed to support limited development of human myeloid cells and often accompanied by a high proportion of immature B lineage. In contrast, NSG‐SGM3 mice have been noted to enhance the generation of human myeloid cells and increased levels of NK cells, along with reconstitution of T and B cells. NSBGW mice that lack endogenous Kit function impairs endogenous mouse HSCs, allowing engraftment of human HSCs without irradiation. This mouse model provides better human erythropoiesis and platelet formation compared to NSG mice. Over the years, researchers have reported the differences in the engraftment of human cord blood cells in recipient male and female immunodeficient mice.^8^, ^9^, ^10^ In addition, the frequency and lineage commitment of the human transplanted HSCs between the published studies have shown different outputs. For example, some studies have estimated that 0.001% to 0.0001% of gene‐corrected CD34^+^ HSCs have the long‐term engraftment potential while others have indicated 0.007%–0.2% of umbilical cord blood (UCB) CD34^+^ HSCs produce engraftment. Variation in the level of engraftment and lineage commitment of xenotransplanted HSPCs have been associated with the donor source.^11^, ^12^, ^13^, ^14^ However, these studies have not considered the impact of donor–recipient sex (mis)match on the outcome of xenotransplantation.

Here, we have used different immunodeficient mouse models, in combination with next‐generation sequencing (NGS) and flow cytometry, to assess the engraftment of hematopoietic cells from AML patients and healthy UCB donors. After noticing trends in our initial results, we went on to investigate the effect of variation in donor and recipient sex on engraftment. Using 38 primary AML patients, we first demonstrate that NSG‐SGM3 (NSGS) mice provide a better model system for studying AML engraftment. Both NSG and NSGS mice are able to maintain long‐term engraftment of leukemia cells with the mutational clonal profile largely conserved in the mouse models. However, we identified engraftment levels specific to the sex of both the donor patient and recipient immunodeficient mouse, which were maintained across different mouse models. The dependence of engraftment on donor–recipient sex categories was also seen when using healthy HSCs from UCB donors. Here, significant differences were also observed in the lineage output. We demonstrate the importance of matching the sex of donors and recipients in xenotransplantation assays. This could explain discrepancies in previous literature on xenotransplantation and has important implications for the future design of preclinical models.

## RESULTS

### NSGS mice enable superior engraftment of AML patient cells irrespective of the disease subtype

**Figure 1:**
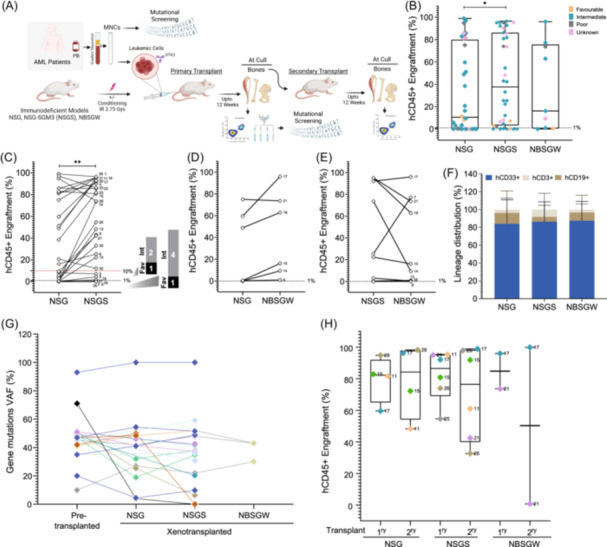
Engraftment levels of primary patient acute myeloid leukemia (AML) cells across various immunodeficient mouse models. (A) Schematic representation of the experimental protocol used to generate primary and secondary xenotransplants using various immunodeficient mouse models. Illustration was created with BioRender.com. (B) Comparison of AML hCD45^+^ cells engraftment in NSG (patients, *n* = 32; mice = 155), NSGS (patients, *n* = 37; mice = 145) and NBSGW mice (patients, *n* = 9; mice = 43). Symbol colors represent AML prognostic categories, for example, favorable, intermediate, and poor‐risk. (C) Paired‐wise analysis of AML patient samples (*n* = 30) transplanted in NSG and NSGS mice. (D) Paired‐wise analysis of AML patient samples (*n* = 6) transplanted in NSG and NBSGW mice. (E) Paired‐wise analysis of AML patient samples (*n* = 9) transplanted in NSGS and NBSGW mice. (F) Lineage distribution within the hCD45^+^ cells in NSG (patients, *n* = 32; mice = 155), NSGS (patients, *n* = 37; mice = 145) and NBSGW (patients, *n* = 9; mice = 43) mice transplanted with AML cells. (G) Mutational spectrum of the AML cells in pretransplanted cells and posttransplanted cells from NSG, NSGS, and NBSGW mice. Each point represents the VAF from cells recovered from one mouse. VAF, Variant allele frequency. Color of the symbol and line represents the mutation across the samples for each gene. (H) Percentage of total hCD45^+^ cells engrafted in primary and secondary transplantation assays in NSG, NSGS, and NBSGW mice. Colored symbols represent same patients transplanted in various mice. (B, C, D, E, H) Each point represents the mean engraftment levels across multiple mice for each patient. Numbers in the panel H correspond to the patient sample used (see Table 1 for reference). Dotted black lines within the panel along *Y*‐axis represents ≥1% hCD45^+^ engraftment. *p < 0.05, **p < 0.001.

Despite the successes seen over the last decade in engrafting primary leukemic cells from AML patients in NSG mice, substantial number of patients fail to regenerate in immunodeficient hosts.^10^ Therefore, we used our NSG, NSGS, and NBSGW mouse models to test 38 primary AML patients (favorable, *n* = 4; intermediate‐risk, *n* = 24; poor‐risk, *n* = 3; unknown, *n* = 7) (Figure 1A and Tables S1 and S2). Available whole‐exome sequencing (WES) or targeted mutation screening data demonstrated the myeloid‐related gene mutational distribution (Table S3) of our cohort was similar to published reports.^15^

Leukemic cells from AML patients were transplanted in NSG (patients, *n* = 32; mice = 155), NSGS (patients, *n* = 37; mice = 145) and NBSGW mice (patients, *n* = 9; mice = 43) (Figure 1B and Figure S1A–B) mice. Overall, AML hCD45^+^ cell engraftment in NSGS mice was higher compared to the NSG and NBSGW mouse models (NSG, median CD45^+^ = 12.65%; NSGS, median hCD45^+ ^= 50.80%; NBSGW, median hCD45^+ ^= 20.45) (Figure 1B). This superior engraftment in NSGS mice was observed more prominently in pair‐wise analysis for each patient (Figure 1C). The patient samples (*n* = 7/15) that generated lower engraftment (hCD45^+^ ≤10%) in NSG mice, engrafted better (median hCD45^+^ 43%) in NSGS mice. Notably, for 8/15 patient samples associated with hCD45^+^ levels ≤10% in NSG mice, engraftment was also poor in NSGS mice (Figure 1C), indicating that the NSGS BM microenvironment is not sufficiently supportive for all AML samples. Patient samples that generated medium/high engraftment (hCD45^+^ ≥40%) in primary transplants in NSG mice performed similarly in the NSGS settings.

NBSGW mice exhibit enhanced engraftment of human hematopoiesis when compared to nonconditioned NSG mice as previously reported.^16^ Based on the observed engraftment levels of AML patient samples (nonengrafters ≤1% and engrafters ≥1%) in NSG and NSGS mice, we selected a number of AML patient samples (*n* = 9) and transplanted them into the NBSGW mice. Four patients (AML10 and AML14, AML17 and AML19) showed improved engraftment in NBSGW mice compared to the NSG, while no difference was observed in the remaining two (AML8 and AML21, Figure 1D). On the other hand, when compared to NSGS, NBSGW mouse model seemed to be less efficient (Figure 1E). Three (AML14, AML19, and AML21) out of the nine patient samples tested engrafted better in NSGS mice compared to NBSGW. Only one patient (AML7) showed significantly improved engraftment in NBSGW compared to the NSGS mice (median hCD45^+^ 22.4% vs. 84.8%). Two patient samples (AML17 and AML10) exhibited similar engraftment levels to those observed in NSGS mice, while the remaining three (AML8, AML12, and AML38) failed to engraft in NBSGW mice, mirroring the outcomes observed in NSGS mice. No differences were observed in the lineage output of the AML patient cells engrafted in all three mouse models, as majority of the cells comprised of myeloid CD33^+^ lineage (Figure 1F). Considering the limited number of patients tested in NBSGW mice, additional independent experiments are necessary to validate these findings in future studies.

The AML samples, we describe in this study, represent a broad spectrum of AML cases from all prognostic risk groups and mutation categories (Figure 1B and Figure S1B–H). In agreement with the previous reports,^17^ favorable risk group tends to generate little or no engraftment in both NSG (patients, *n* = 5) and NSGS (patients, *n* = 3) mouse models. Among the intermediate‐risk patients, 40% (9/22 patients) in NSG and 61% (14/23 patients) in NSGS generated high (hCD45^+^ ≥20%) engraftment levels. Poor risk AML patients were likely to be high engrafters (2/3 patients) in both NSG, as well as NSGS mice (Figure S1C–D). NPM1^Mut^‐FLT3^WT^ and NPM1^WT^‐FLT3^WT^ patients generated lowest engraftment in the NSGS mice while no differences were observed in the NSG model (Figure S1E–F).

It is important to access the clonal identity of the human cells engrafted in mice to confirm the maintenance of leukemic genotype. Therefore, we used NGS platform to compare the relevant mutations present in the engrafted cells in the mice with those that were initially identified at diagnostic stage. Variant allele frequency (VAF) of the gene mutations was largely recapitulated in the matched PDX samples across all the mouse models tested (Figure 1G and Table S4). However, in certain cases, fluctuations were observed in some of the mutations. Therefore, it is crucial to verify the status of the engrafted clones before proceeding with any downstream analysis. Overall, our data confirms that all mouse models used here were able to maintain the original leukemic clone(s) in the patient‐derived xenografts (PDXs).

### NSG and NSGS mice are both able to maintain long‐term potential of patients' leukemia‐initiating cells

In comparison to NSG and NBSGW mice, NSGS model has been shown to cause the exhaustion of the healthy hematopoietic stem and progenitor cells (HSPCs).^18^ Therefore, we tested the long‐term initiating capacity of the AML patient cells (patients, *n* = 6; mice, *n* = 49) by performing the secondary transplantation (NSG > NSG, NSGS > NSGS, NBSGW > NBSGW) in all the three mouse models (Figure 1H). No significant differences were observed in the engraftment of the leukemic cells in the secondary transplants between the mouse models (median hCD45^+^ NSG = 76.44%, NSGS = 84.22% and NBSGW = 50.32%). Only AML21 yielded lower engraftment in the NBSGW mice compared to the NSGS model (mean hCD45^+^ NSGS = 42.5% vs. NBSGW = 0.76%). All the cells engrafted in the secondary transplants were of myeloid lineage across all the mouse models (Figure S1I). The ability of leukemic initiating cells (LICs) to persist in secondary NSGS mice, in contrast to normal HSCs, may suggest a distinctive feature of leukemia itself. LICs are generally considered to possess a greater capacity for self‐renewal and longevity, potentially making them closer to a state of immortality compared to their normal HSCs counterpart, and therefore our results are not necessarily surprising.

### Primary patient AML cells and recipient immunodeficient mice demonstrate sex‐specific engraftment potential

**Figure 2:**
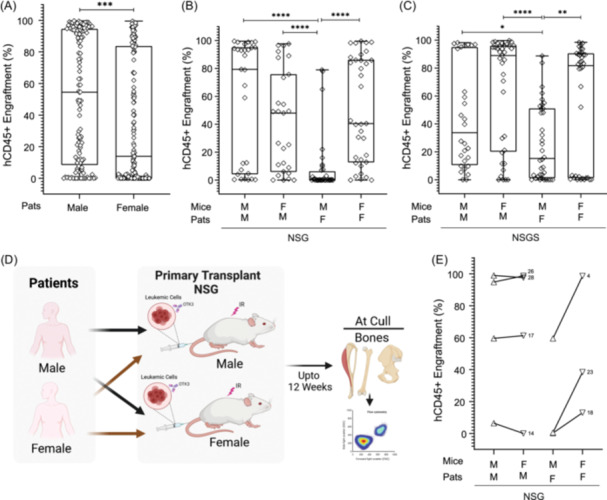
Sex matching of primary patient AML cells and recipient mouse is essential for generating higher engraftment levels. (A) AML hCD45^+^ cells engraftment based on the sex of the AML patients (*n* = 38) in NSG and NSGS mice. (B) AML hCD45^+^ cells engraftment based on the sex of the AML patients (*n* = 28) and the recipient NSG mice (*n* = 127). (C) AML hCD45^+^ cells engraftment based on the sex of the AML patients (*n* = 35) and the recipient NSGS mice (*n* = 137). (D) Schematic representation of the experiment plan. Illustration was created with BioRender.com. (E) Paired‐wise analysis of AML patient samples (*n* = 7) transplanted in NSG male and female mice (*n* = 56). Data are presented as mean engraftment for each patient. For (A–C), each data point represents one mouse. F, female; M, male; Pats, patients. *p < 0.05, **p < 0.001, ***p < 0.0001, **** *p* < 0.00001.

The impact of biological sex on incidence, prognosis, and mortality has been shown in a wide range of cancers, unrelated to reproductive function.^19^ We hypothesized that the engraftment capacity of human cells or tissues in mice may be influenced by some of these clinical factors. In order to understand the effect of patients' sex on engraftment potential, we incorporated donor sex as a variable in our analysis, aiming to elucidate its role in engraftment levels. Interestingly, female patients generated significantly lower human cell output compared to the male counterparts (median hCD45^+^ 13.9% vs. 54.5%), irrespective of the disease subtype and mouse model (Figure 2A and Figure S2A–C). The effect of patients' biological sex on the level of human cell engraftment was pronounced when sex of the recipient mice and donor patients were matched, particularly for the female–female group (Figure 2B,C). Female patient cells transplanted into the male recipient mice yielded little or no engraftment, while as in female recipient mice, human cell engraftment of female patients was significantly higher (median hCD45^+^ 0.19% vs. 40.5%). Interestingly, male patient cells engrafted significantly better in both male and female recipient mice (median hCD45^+^ 33.6% and 48%). This recipient–donor sex‐specific engraftment capacity of AML patients was observed in both NSG, as well as NSGS mice (Figure 2B,C) and was not driven by the AML prognostic risk groups (Figure S2D–E).

Next, we decided to transplant AML patient (*n* = 7) cells into NSG mice with a focus on matching/mismatching recipient and donor sex (Figure 2D). We included AML patients that were categorized as low engrafters (hCD45^+^% ≤ 10%), as well as high engrafters (hCD45^+^% > 10%) in this analysis (Figure 1B). Interestingly, no significant differences were observed for male AML patients when transplanted into male or female recipient mice (Figure 2E). However, female donor AML cells when transplanted into the female recipient mice generated significantly higher engraftment compared to the male recipient mice (Figure 2E).

### Healthy human hematopoietic stem/progenitor cells in immunodeficient mice behave in sex‐specific manner

**Figure 3:**
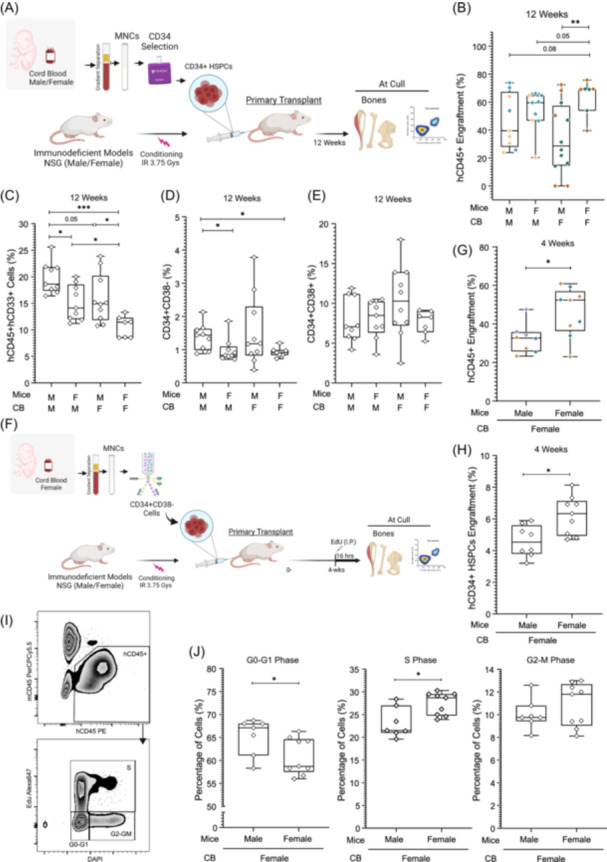
Healthy umbilical cord blood (UCB) cells from male and female donors perform differently based on the sex of the recipient mice. (A) Schematic representation of the experimental protocol used to generate primary xenotransplants using NSG mouse model. Illustration was created with BioRender.com. (B) hCD45^+^ cells engraftment based on the sex of the UCB cells and the recipient NSG mice at Week 12. Two independent pooled donors from male and female UCBs were used. Each colored symbol represents a pool of donors. (C) Percentage of hCD33^+^ within the hCD45^+^ cells in NSG mice transplanted with healthy UCB cells at Week 12. (D) Percentage of CD34^+^CD38^−^ stem cells in mice transplanted with female or male UCB cells injected into either male or female mice at Week 12. (E) Percentage of CD34^+^CD38^+^ progenitor cells in mice transplanted with female or male UCB cells injected into female or male recipient mice at Week 12. (F) Schematic representation of the experimental protocol used to access human cell proliferation in primary NSG mice. Illustration was created with BioRender.com. (G) Female healthy UCB cells engraftment (% hCD45) in NSG male and female mice at Week 4, four independent pooled donors from female UCBs were used. (H) Percentage of CD34^+^ HSPCs following the xenotransplantation of female healthy UCB cells in NSG male and female mice at Week 4 (I) Representative FACS plot of human CD45 cell engraftment and cell cycle profile of these cells following the injection of EDU in NSG mice at Week 4. (J) Cell cycle profile of female UCB hCD45^+^ cells engrafted in NSG male and female mice at Week 4. Each point represents one mouse transplanted with healthy UCB cells. *p < 0.05, **p < 0.001, ***p < 0.0001. UUCBCB, umbilical cord blood cells; F, female; M, male.

Given our leukemic patient data demonstrated recipient–donor sex bias in engraftment, we decided to understand if this behavior was also observed with healthy donor cells. HSPCs (CD34^+^) derived UCB cells from male and female donors were transplanted into male and/or female NSG mice (Figure 3A). We used cells from pool (each pool contained at least 3 UCB donors) of UCB donors of the same sex to perform these experiments. Mice were sacrificed 12 weeks after transplantation of human cells. Unlike AML patient samples, male and female UCB donor cells generated similar average engraftment levels in NSG mice (Figure S3A). However, this masked a significant difference in the engraftment of female donor cells between male and female recipients (Figure 3B). In fact, female HSPCs in female recipient mice seemed to perform better than any other combination, although this was not statistically significant (Figure 3B). We also noticed higher variability of human grafts in male‐recipient mice across independent experiments performed here (Figure 3B).

### Lineage and stem cell output of healthy human hematopoietic cells is highly based on the sex of both donor cells and recipient mice

Next, we assessed whether recipient donor sex had any effect on the lineage output of the donor HSPCs. NSG mice have been shown to be biased toward producing lymphoid B‐cell (hCD45^+^hCD19^+^) lineage when transplanted with human UCB cells.^20^ B‐cell output from female donor HSPCs was similar in male and female recipient mice (Figure S3B). We also observed a significant increase in myeloid (hCD45^+^CD33^+^) cells from UCB male donor into male recipient (Figure 3C and Figure S3B) mice.

The proportion of engrafted human hCD45^+^hCD34^+^hCD38^−^ that is known to contain the long‐term HSCs after 12 weeks of xenotransplantation were significantly lower (mean = 0.92%) in female–female compared to male–male (mean = 1.38%) groups (Figure 3D). Interestingly, engrafted hCD45^+^hCD34^+^hCD38^−^ HSPCs from male donors were significantly higher in proportion in male recipient compared to the female recipient mice. On the other hand, no significant differences were observed in proportion of hCD45^+^hCD34^+^hCD38^+^ hematopoietic progenitors between the female–female and male‐male groups (Figure 3E).

### Female human HSCs proliferate better in female mice but have reduced stem cell maintenance

Quantification of the proliferative capacity and life span of HSCs is fundamental for understanding recipient–donor effect, population levels, and cellular homeostasis in xenograft models. One of the best approaches to accomplish this goal is to directly label nascent DNA using EdU (5‐ethynyl‐2′‐deoxyuridine) in in vivo. Therefore, we transplanted FACS‐isolated CD34^+^CD38^−^ female UCB cells into male and female NSG mice. Four weeks following the transplantation, mice were injected with EdU and then 16 h later mice were sacrificed (Figure 3F). Consistent with our 12‐week data, hCD45^+^ cell engraftment in female–female group was significantly higher compared to the male mice that received female healthy donor cells (Figure 3G). Human CD34^+^ HSPCs were significantly higher in female–female compared to the male mice (Figure 3H). Interestingly, this was driven by CD34^+^CD38^+^ cells and not CD34^+^CD38^−^ population (Figure S3C–D) which could be due to the fact that progenitor cells are driving the engraftment, at this early stage.

To determine the proliferation and cell cycle status of in vivo EdU‐labeled human‐engrafted cells, we performed FACS analysis (Figure 3I) of these cells. Healthy donor female hCD45^+^ cells in male recipient mice were present in higher proportions in G0–G1 phase compared to the female–female group (Figure 3J). In contrast, healthy donor female cells in female mice were proliferating at a higher rate compared to the male mice, as significantly more cells were detected in S‐phase of the cell cycle. Although female donor cells in female mice were also higher in G2–M phase compared to the male mice, this was not statistically significant (Figure 3J). This data is consistent with the higher engraftment levels observed for female donor cells in the female–female group. Altogether, our data demonstrates that sex matching of donor–recipient plays an essential role in xenotransplantation assays and that female donor cells are more sensitive to the sex of the recipient mice for their engraftment.

## DISCUSSION

Previous published work studying HSPC engraftment in immunodeficient mice have demonstrated the superior engraftment of human cells in female recipient mice, and this has been linked to the presence of higher levels of estrogen.^9^, ^21^ However, it is noteworthy that these studies have not comprehensively investigated the potential influence of donor sex on generating successful transplants. This aspect is crucial to consider, as emerging data suggests that the biological and immunological differences associated with donor‐recipient sex could potentially play a significant role in the outcome of such assays.^6^, ^22^ In line with this, our findings reveal a new dimension of xenotransplantation, whereby matching donor–recipient sex is essential in generating a reliable and potentially, biologically relevant xenotransplantation model.

An initial observation showed significantly superior engraftment of the leukemia cells from the male patients compared to the female counterparts in male recipient mice. Further investigation revealed that when donor cells and recipient mice are sex‐matched, there is no significant difference between male and female AML patient engraftment. Intriguingly, a negative impact on the engraftment of female leukemia patient cells was observed where male‐recipient mice were used for transplantation. In NSG mice, there was indeed little or no engraftment of the female AML donor cells in male mice while this effect was marginally improved in NSGS model probably as a result of the three human cytokines playing a positive role. This suggests that matching the sex of donor and recipient may offset the observed sex‐related disparities in AML engraftment to some extent. While NSGS mice have demonstrated improved engraftment of AML cells in general, a similar behavior was observed in NSG mice when donor‐recipient sex was matched for female patients. An adverse impact on the engraftment of female leukemia cells in male‐recipient mice raises intriguing questions about the interplay that may exist between sex‐specific factors and the host environment at least in the context of AML. It is worthy to note that male NSG mice appear to serve as more suitable recipients for male leukemia cells, whereas within the NSGS strain, female recipients exhibit enhanced compatibility with male grafts.

Although the engraftment of healthy donor cells exhibited similarity between male and female donors, a striking difference was seen when female healthy donor cells were transplanted in male mice. In contract to AML patient cells, female healthy donor cells demonstrated the ability to generate grafts in the male mice albeit at a lower level compared to sex‐matched female counterpart. In general, female healthy donor cells in male recipient mice tend to have significantly lower engraftment compared to all the donor‐recipient transplant combinations. The contrasting behavior of healthy donor cells and AML suggests that the influence of donor sex on engraftment levels may be an additional interplay between the leukemic cells and the host microenvironment. In the clinical settings, there is no evidence to suggest that the outcomes of hematopoietic stem cell transplantation (HSCT) in human patients significantly differ between male and female patients. However, there is an increased risk of chronic/acute graft‐versus host disease (GvHD) in recipients of grafts from female donors. Interestingly, the effect of the recipient sex in GvHD and relapse is still not entirely clear with contradictory studies published over the years.^23^, ^24^, ^25^, ^26^

Studies using mouse models have uncovered critical differences in hematopoiesis,^27^, ^28^, ^29^ particularly the influence of sex hormones, such as estrogen, in promoting HSC proliferation, as well as self‐renewal capacity, which leads to augmented erythropoiesis.^30^ Our investigation aligns with these findings, revealing noteworthy cell cycle levels for female healthy donor cells in xenotransplantation. Specifically, female donor cells when transplanted into female recipient mice have an increased proportion of cells in the S‐phase and a decrease in the G0–G1 phase. Interestingly, more CD34^+^CD38^−^ HSCs were present in male recipient mice when transplanted with female healthy donor cells compared to female mice, although this difference was not significant. Male–male sex xenotransplantation combination maintained higher proportions of CD34^+^CD38^−^ donor HSCs compared to the female–female counterparts. A study by Illing et al. has demonstrated that mouse HSPCs when treated with estradiol appear to become exhausted sooner in serial transplantation experiments.^31^ Furthermore, it is well established that women consistently exhibit a lower incidence of hematologic cancers compared with men. This raises important questions, for example, given the increase in the cell cycle activity of the HSPCs due to elevated estrogen levels, what intrinsic or extrinsic regulatory mechanisms come into play to govern aging processes? These mechanisms may play a critical role in preventing the eventual depletion or exhaustion of these cells. Although it is highly plausible that the primary female sex hormone estrogen plays a role in hematopoiesis, it is unlikely that estrogen is the only pivoting factor driving this sex‐based disparity. There could be additional microenvironment‐related factors that have a significant bearing on the regulation of hematopoiesis and highlight the complexity of sex‐related interactions in such processes, which could ultimately have a bearing on the human HSCT in the clinics. Recently, female donor/male recipient (FDMR) mismatching has been included in the European Group for Blood and Marrow Transplantation (EBMT) risk scoring system for transplantation considerations. It has been shown that FDMR mismatch predicts an increased risk of adverse outcomes following HSCT as compared to sex‐matched donor–recipient pairs.^32^, ^33^

In conclusion, our findings underscore the importance of carefully considering and reporting the sex of donor and recipient pairs in xenograft studies. Moving forward, strict adherence to sex‐specific matching protocols will be imperative for the accurate interpretation and advancement of xenotransplantation models, particularly to guide new paths for research into clonal hematopoiesis and hematological disorders. It is important to note that addressing the underrepresentation of women's health in research has been a longstanding concern.^34^ Our data highlights the importance of increasing the inclusion of female donor samples in pre‐clinical research, as well as clinical trials to enhance the comprehensiveness and relevance of findings. Furthermore, elucidating additional sex‐specific mechanisms of regenerative hematopoiesis has also implications for thousands of HSCT transplantations that are performed across the world. By deepening our understanding of intrinsic and extrinsic factors regulating sex‐based hematopoiesis and HSC function, we have enormous potential to expand and refine the utility of HSCT.

## METHODS

### Patient samples and UBC cells

UCB and AML patient PB samples were received from the Barts Health NHS Trust. All patients had provided written informed consent in accordance with local tissue bank guidelines. Both UCB and AML projects were approved by the East London Ethical Committee and carried out in accordance with the Declaration of Helsinki. Patients' clinical characteristics are detailed in Table S1. Clinical variables for all patients were determined at the time of sample collection. WES data or myeloid‐associated gene panel data (Tables S1–S3) was used to access the baseline clonality of the primary AML patient samples. For WES data, we only focussed on the somatic gene mutations that have been previously associated with AML, for example, DNMT3A, ASXL1, TET2, ETV6, NPM1, RUNX1, TP53, FLT3, IDH2, SRSF2, NRAS, BCOR, IDH1, NF1, STAG2, GATA2, PTPN11, U2AF1, WT1, EZH2, PHF6, CEBPA, KRAS, CREBBP, JAK2, NOTCH1, ATM, RAD21, CBL, HRAS, KIT, MSH6, PDGFRB, SMARCB1, IKZF1, MYC, NTRK1, SMARCA4, ZRSR2, CSF3R, SMC3, FGFR3, RET, ERBB2, SMC1A, MPL, BRAF, PDGFRA, MYD88, PTEN, JAK3, ABL1, and SF3B1.

### Mononuclear cell (MNC), CD34^+^ isolation from the human samples

MNCs from UCB and AML PB samples were isolated by density centrifugation using Ficoll‐Paque TM PLUS (GE Healthcare Life Sciences). Following on, red blood cell lysis was performed using ammonium chloride solution by incubating the cells at 4°C for 10 min.

CD34^+^ cell enrichment for UCB samples was performed using EasySep Human CD34 Positive Selection Kit II (StemCell Technologies) according to the manufacturer's instructions. For UCB experiments, individual donors were pooled together based on the sex of the donors.

### Cell sorting

UCB CD34^+^ enriched cells were stained with antibodies specific for human stem cell antigens (hCD34, hCD38, hCD45RA, and hCD90; BD Biosciences). 4′,6‐diamidino‐2‐phenylindole (DAPI, 1:1000 dilution from a 200 μg/mL stock) dye was used to discriminate between live and dead cells. Cell sorting was performed using a FACS Aria SORP (BD Biosciences). Following the exclusion of nonviable cells, debris, and doublets, CD34^+^CD38^−^ cells were sorted. Then cells were counted to attain accurate cell count before injecting them into the mice.

### Xenotransplantation

NOD/SCID/IL2rγ−/−(NSG) and NOD/SCID/IL2rγ−/−/IL‐3/GM/SF (NSG‐SGM3, NSGS) mice were originally obtained from Leonard Shultz (The Jackson Laboratory). NOD/SCID/IL2rγ−/−/Tyr+/Kit W41J (NBSGW) mice were purchased from the Jackson Laboratory. All three strains of mice were bred at the Francis Crick Institute Biological Resource facility. Male and female mice aged between 8 and 12 weeks of age were used and all animal experiments were performed at the Francis Crick Institute in accordance with UK Home Office and CRICK guidelines and were undertaken under the Home Office project license PLL 70/8904.

NSG and NSGS mice received a sublethal dose of radiation (3.75 Gys) from a cesium‐137 source 24–48 h prior to procedure. Mice were maintained on antibiotics for 7 days after the conditioning. T‐cell depletion was performed by treating MNCs with anti‐CD3 mAb OKT3 antibody (BioXCell) prior to injection into the mice as described previously.^35^, ^36^ 5 × 10^6^ AML CD3‐depleted MNCs were injected into the NSG, NSGS, and/or NBSGW mice via the tail vein (see Table S2 for more details). Mice were culled and engraftment were assessed in the BM (pooled femurs, tibias, pelvis) at the time of sacrifice (up to 12 weeks).

For secondary transplantation assays, hCD45^+^ cells from primary transplants were isolated using EasySep™ Release Human CD45 Positive Selection Kit (StemCell technologies). Selection of human xenografted cells from primary transplants was only performed if the engraftment of human CD45^+^ cells was <90% in the primary transplanted mice. For primary transplants where engraftment of human cells was ≥90%, total xenografted cells from primary mice were directly injected into the secondary mice.

UCB CD34^+^ cells (50,000 cells) or CD34^+^CD38^−^ cells (10,000 cells) from male and/or female donors were injected into the NSG mice via the tail vein following the irradiation. Mice were culled and engraftment was assessed in the BM (pooled femurs, tibias, iliac crests) at the time of sacrifice (CD34^+^CD38^−^, 4 weeks or CD34^+^, 12 weeks).

### In vivo EdU cell proliferation assay

NSG mice (male and female) were injected (intraperitoneal route) with 200 μL of 10 mM EdU at Week 4 following the transplantation of male or female UCB CD34^+^CD38^−^ cells. Sixteen hours following the EdU injection, mice were culled and BM (pooled femurs, tibias, pelvis) was recovered. Red blood cell lysis was performed using ammonium chloride solution by incubating the cells at 4°C for 10 min. EdU staining was conducted using Click‐iT™ Plus EdU Flow Cytometry Assay Kit (Invitrogen) according to the manufacturer's protocol.

### Flow cytometry analysis and cell sorting for xenografted samples

Cells recovered following culling of the mice were stained with antibodies specific for human or murine antigens (differentiation panel: mCD45, hCD45, hCD33, hCD19, and hCD3; stem cell panel: mCD45, hCD45, hCD34, hCD38, hCD45RA, and hCD90; BD Biosciences). DAPI or PI (propidium iodide) dye was used to distinguish between live and dead cells during the analysis. Cells were immunophenotyped by using Fortessa flow cytometer (BD Biosciences). Human myeloid (hCD45^+^hCD33^+^), B cells (hCD45^+^hCD19^+^), T cells (hCD45^+^hCD3^+^), HSPCs (hCD45^+^hCD34^+^hCD38^+^, hCD45^+^hCD34^+^hCD38^−^), HSCs (hCD45^+^hCD34^+^hCD38^−^CD45RA^−^CD90^+^), MPPs (hCD45^+^hCD34^+^hCD38^−^CD45RA^−^CD90^−^), and MLPs (hCD45^+^hCD34^+^hCD38^−^CD45RA^−^) were analysed as individual cell populations.

Cell sorting was performed using a FACS Aria SORP (BD Biosciences). Cells were sorted as follows, myeloid cells (mCD45^−^hCD45^+^hCD33^+^), lymphoid T cells (mCD45^−^hCD45^+^hCD3^+^), lymphoid B cells (mCD45^−^hCD45^+^hCD19^+^). Sorted cells were washed in phosphate‐buffered saline and harvested in order to later perform further mutational analysis, where needed.

### DNA‐targeted mutation analysis

Genomic DNA extraction from FACS‐sorted xenografted cells was performed using DNeasy Blood & Tissue Kit (Qiagen). Targeted gene mutation screening was performed as previously described.^37^ Briefly, oligos for patient‐specific mutations were designed using Primer3 program for performing polymerase chain reaction. All mutation‐specific oligos were designed to exclusively target human DNA, ensuring the generation of products only from human DNA. Then, amplified amplicons were normalized and mixed together based on the mutations/patients. Transposon‐based Nextera technology (Illumina) was then used to prepare illumine libraries. Amplicon libraries were sequenced on the Illumina MiSeq platform. Variant Studio (Illumina) and integrated genome viewer were used to visualize VCF and BAM data files, respectively.

### Statistical analysis

Statistical analysis of the data was performed using Prism Version 6 software (GraphPad). Data is presented as the mean ± SEM (where applicable). Mann–Whitney *U* test or Wilcoxon matched‐pairs signed rank test (where applicable) was used to compare the data points. All the significant *p* values are described in the legends of the figures (where applicable).

## AUTHOR CONTRIBUTIONS

Syed A. Mian and Linda Ariza‐McNaughton performed the research, analyzed data, and wrote the manuscript. Fernando Anjos‐Afonso and Remisha Guring performed the experiments. Sophie Jackson and Aytug Kizilors performed molecular analysis and analyzed results. John Gribben provided AML samples and relevant patient information. Dominique Bonnet supervised research, analyzed data and reviewed the manuscript. All authors approved the manuscript.

## CONFLICT OF INTEREST STATEMENT

The authors declare no conflict of interest.

### FUNDING

This works was supported by the Francis Crick Institute which receives its core funding from Cancer Research UK (CC1045), the UK Medical Research Council (CC1045), the Wellcome Trust (CC1045) to D. B.

## Supporting information

Supporting information.

Supporting information.

Supporting information.

Supporting information.

Supporting information.

Supporting information.

Supporting information.

Supporting information.

## Data Availability

The data that supports the findings of this study is available in the supplementary material of this article.
