# Correction to “Influence of donor–recipient sex on engraftment of normal and leukemia stem cells in xenotransplantation”

**DOI:** 10.1002/hem3.119

**Published:** 2024-07-19

Mian S, Ariza‐McNaughton L, Anjos‐Afonso F, et al. Influence of donor–recipient sex on engraftment of normal and leukemia stem cells in xenotransplantation. *HemaSphere*. 2024;8:e80.

In the author listing of the manuscript, the last name of an author was misspelled. The correct spelling is Remisha Gurung. This has been corrected.

We apologize for this error.

