# Supplementary figures and images for: Influence of donor–recipient sex on engraftment of normal and leukemia stem cells in xenotransplantation
Source: Hemasphere. 2024 May 21;8(5):e80. doi: 10.1002/hem3.80 (PMC11107397; doi:10.1002/hem3.80)

Supp Fig 1

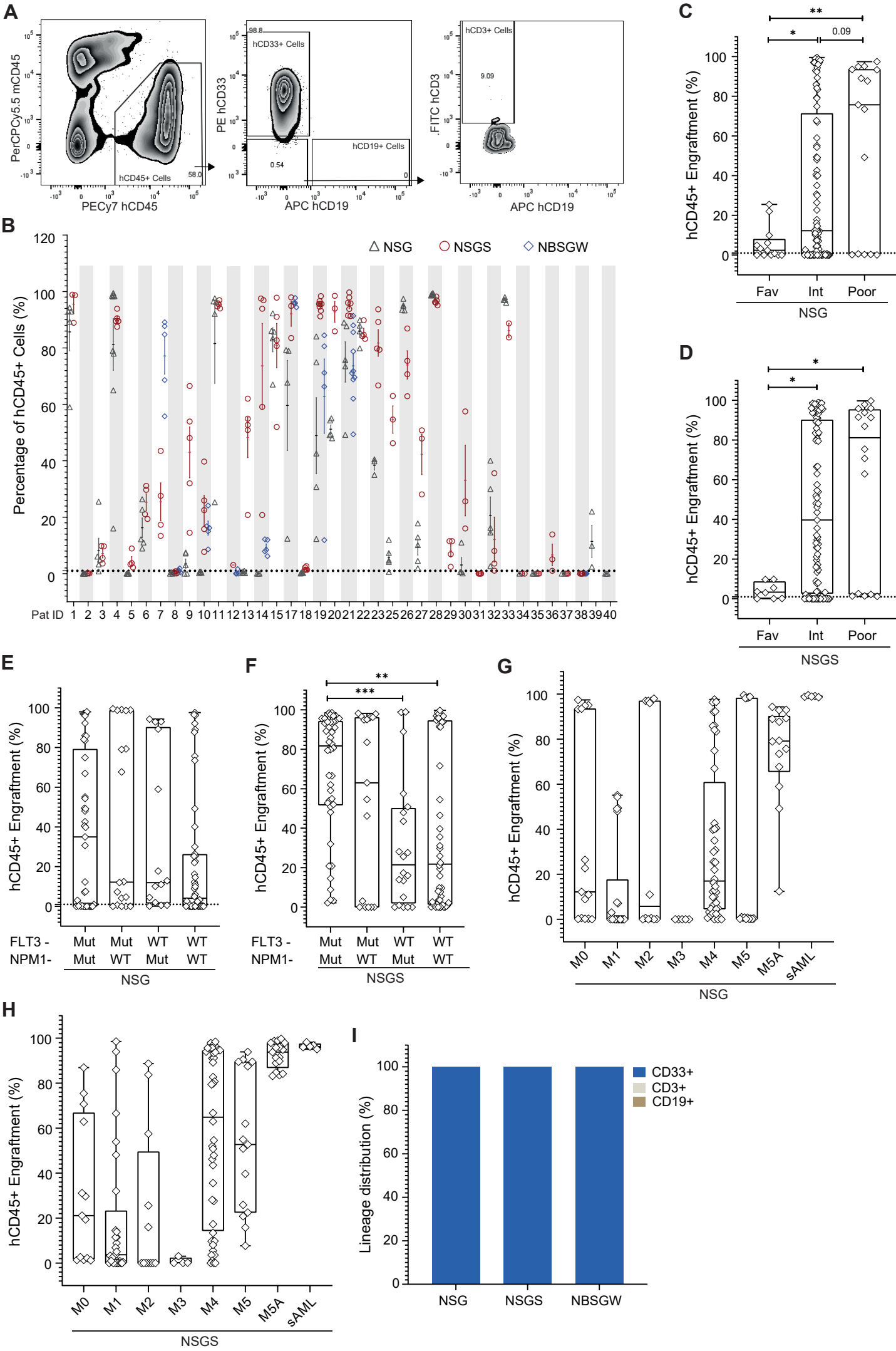

Supplement: Supplementary file 1 — Supporting information. [file HEM3-8-e80-s002.pdf]

Supp Fig 2

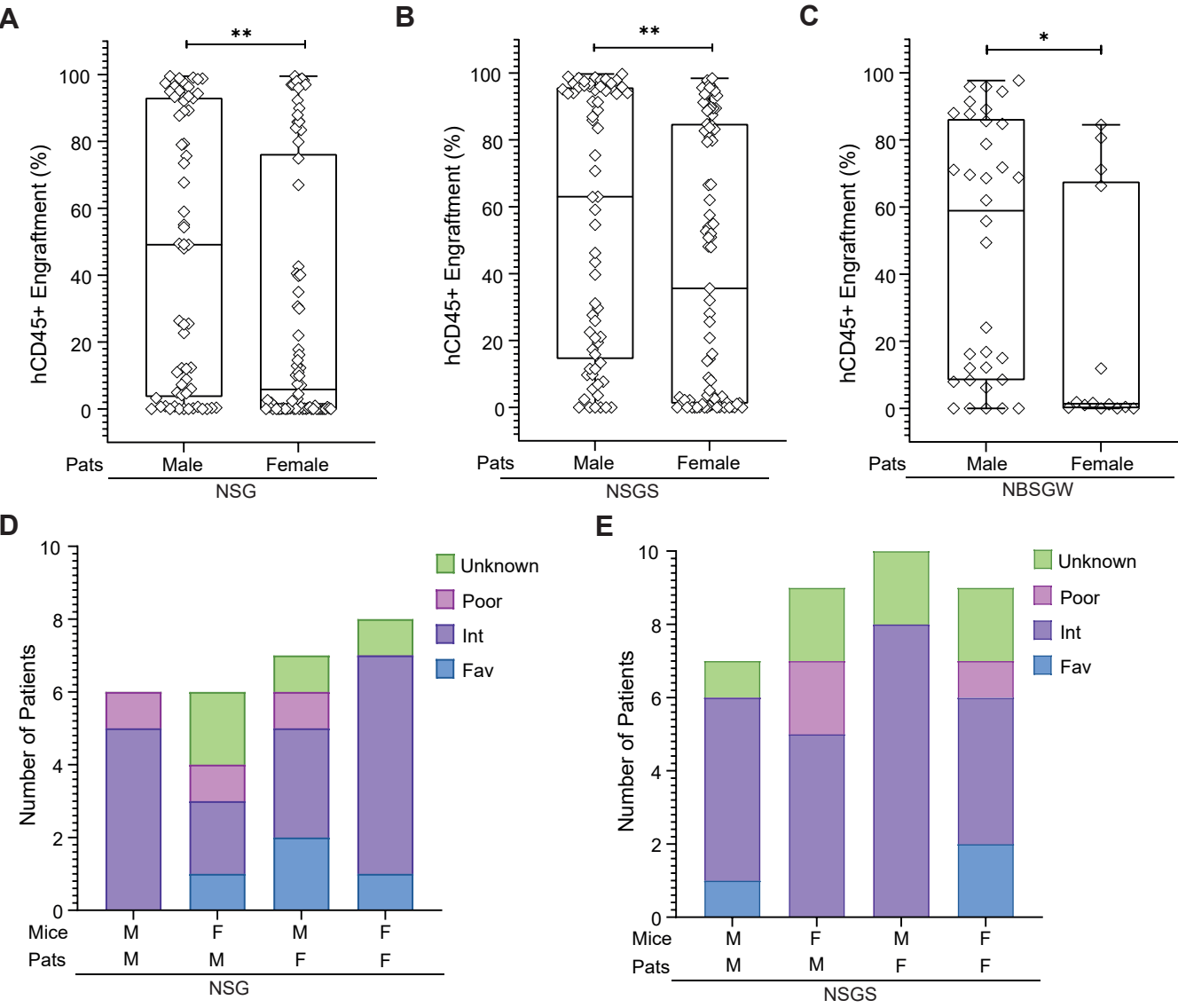

Supplement: Supplementary file 2 — Supporting information. [file HEM3-8-e80-s007.pdf]

Supp Fig 3

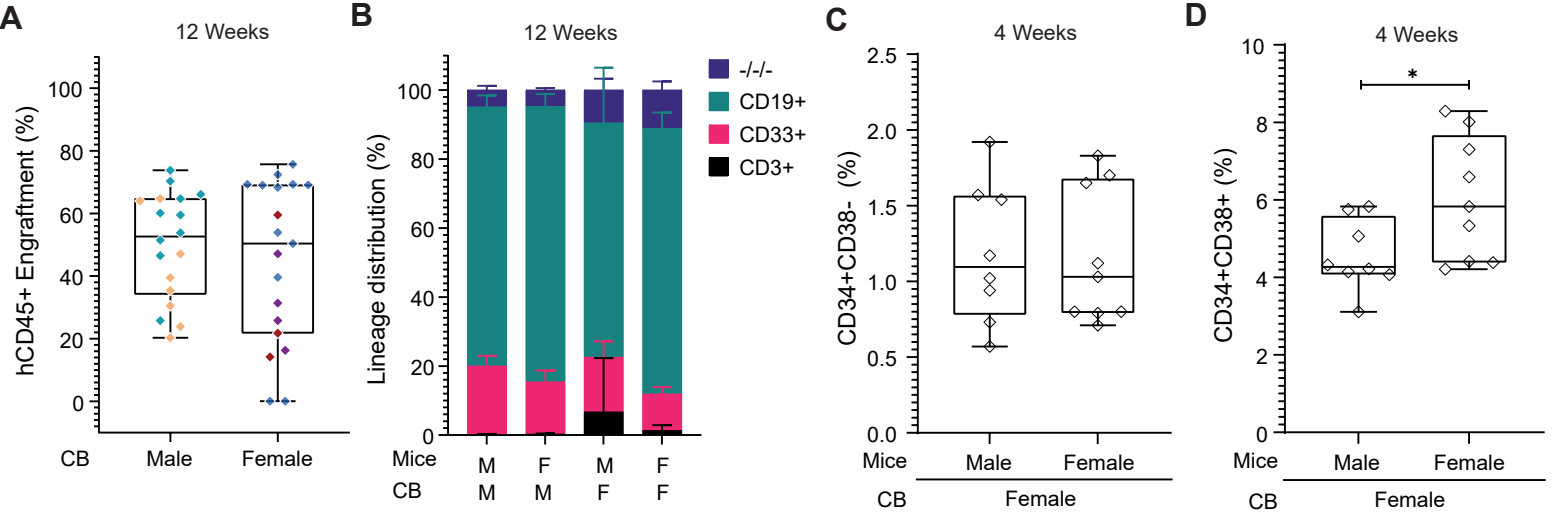

Supplement: Supplementary file 3 — Supporting information. [file HEM3-8-e80-s003.pdf]
