# Supplementary material for: Influence of donor–recipient sex on engraftment of normal and leukemia stem cells in xenotransplantation
Source: Hemasphere. 2024 May 21;8(5):e80. doi: 10.1002/hem3.80 (PMC11107397; doi:10.1002/hem3.80)
Supplement: Supplementary file 8 — Supporting information. [file HEM3-8-e80-s006.docx]

**Supplementary figure legends**

**Figure S1: hCD45 engraftment levels of primary patient AML cells in NSG, NSGS and NBSGW immunodeficient mouse models.** **(A)** Representative FACS plot of human CD45 cell engraftment and gating strategy of immunodeficient mouse at cull. **(B)** Primary patient AML hCD45^+^ cells engraftment in NSG, NSGS and NBSGW mice. Each point represents one mouse. **(C) (D)** Comparison of total hCD45^+^ cell engraftment based on AML prognostic categories e.g. favorable, intermediate and poor-risk, in NSG and NSGS mouse models, respectively. **(E)** Comparison of total hCD45^+^ cell engraftment based on the base-line mutation profile of AML patients in NSG mice **(F)** Comparison of total hCD45^+^ cell engraftment based on the base-line mutation profile of AML patients in NSGS mice **(G)** Comparison of total hCD45^+^ cell engraftment based on AML WHO FAB classification in NSG mice. **(H)** Comparison of total hCD45^+^ cell engraftment based on AML WHO FAB classification in NSG mice. (I) Lineage distribution within the hCD45^+^ cells of AML samples engrafted in secondary mice NSG (n=4; mice=22), NSGS (Patients, n= 6; mice=26) and NBSGW (Patients, n=2; mice=7).

**Figure S2: AML patient and recipient mouse sex determines the engraftment levels of the donor cells in xenotransplantation assays (A)** hCD45^+^ cell engraftment of male and female AML patients in recipient NSG mice. **(B)** hCD45^+^ cell engraftment of male and female AML patients in recipient NSGS mice. **(C)** hCD45^+^ cell engraftment of male and female AML patients in recipient NBSGW mice. **(D) (E)** Distribution of patients based on AML prognostic categories e.g. favorable, intermediate and poor-risk, that were engrafted in NSG/NSGS mice and were used to perform sex-based donor-recipient analysis in Fig2B and 2C.

**Figure S3: UCB cells from healthy male and female donors generate variable lineage and HSPC output in recipient mice. (A)** hCD45^+^ cell engraftment of male and female UCB pool in recipient NSG mice at 12 weeks post-transplant. **(B)** Lineage distribution within the hCD45^+^ cells of male or female UCB pool transplanted in either male or female recipients. **(C)** Percentage of CD34^+^CD38^-^ stem/progenitor cells in mice (female or male) transplanted with female UCB pool at week 4. **(D)** Percentage of CD34^+^CD38^+^ progenitor cells in (female and male) mice transplanted with female UCB pool at week 4**.**

**Supplementary table legends**

**Table S1:** Clinical chrematistics of the patient samples that were used in this study. FAB, French-American-British,

**Table S2:** Details of the experiments performed for each patient.

**Table S3:** Somatic mutation profile of the patient cells prior to xenotransplantation. VAF- variant allele frequency.

**Table S4:** Mutation analysis of the pre-transplanted and post-xenotransplanted cells.
